# The Evaluation of a Family-Engagement Approach to Increase Physical Activity, Healthy Nutrition, and Well-Being in Children and Their Parents

**DOI:** 10.3389/fpubh.2021.747725

**Published:** 2021-12-09

**Authors:** Mathilde R. Crone, M. Nienke Slagboom, Anneloes Overmars, Lisa Starken, Marion C. E. van de Sande, Noortje Wesdorp, Ria Reis

**Affiliations:** ^1^Department of Public Health and Primary Care, Leiden University Medical Center, Leiden, Netherlands; ^2^Faculty of Social Work and Education, The Hague University of Applied Science, The Hague, Netherlands; ^3^Amsterdam Institute for Global Health and Development, University of Amsterdam, Amsterdam, Netherlands; ^4^School of Child and Adolescent Health, The Children92s Institute, University of Cape Town, Cape Town, South Africa

**Keywords:** family, engagement, intergenerational, prevention, overweight, psychosocial, social context

## Abstract

Prevention programs often are directed at either parents or children separately, thereby ignoring the intergenerational aspect of health and well-being. Engaging the family is likely to improve both the uptake and long-term impact of health behavior change. We integrated an intergenerational approach into a frequently used shared assessment tool for children's care needs. The current study's aim was 2-fold: to monitor this family-engagement tool's effects on both children and their parents' health behaviors and well-being, and to examine the different dynamics of health behavioral change within a family.

**Method:** We followed 12 children ages 10–14 years and their parents for 12 weeks using an explanatory mixed-methods design comprising interviews, questionnaires, and an n-of-1 study. During home visits at the beginning and end of the study, we interviewed children and their parents about their expectations and experiences, and measured their height and weight. Furthermore, we collected secondary data, such as notes from phone and email conversations with parents, as well as evaluation forms from professionals. In the n-of-1 study, families were prompted three times a week to describe their day and report on their vegetable intake, minutes of exercise, health behavior goals, and psychosomatic well-being. The interviews, notes, and evaluation forms were analyzed using qualitative content analyses. For the n-of-1 study, we performed multi-level time-series analyses across all families to assess changes in outcomes after consulting the family-engagement tool. Using regression analyses with autocorrelation correction, we examined changes within individual families.

**Results:** Five child-mother dyads and three child-mother-father triads provided sufficient pre- and post-data. The mean minutes of children's physical activity significantly increased, and mothers felt more energetic, but other outcomes did not change. In consultations related to overweight, the family-engagement tool often was used without setting specific or family goals.

**Conclusions:** The family-engagement approach elicited positive effects on some families' health and well-being. For multifaceted health problems, such as obesity, family-engagement approaches should focus on setting specific goals and strategies in different life domains, and for different family members.

## Introduction

Changing health behavior within families is a well-known challenge (1, 2). This study evaluates the use of a family-engagement tool to increase physical activity, healthy nutrition, and well-being in children and their parents in Katwijk, the Netherlands. This former fishing village previously was known for its close-knit families and distinct social structure, in which men worked offshore for weeks or months, while women stayed home and took care of their children (3). The community has experienced rapid contextual changes over the past five decades due to welfare reforms, climate change, and globalization (3). Public health data from Katwijk indicates that 21% of 10- and 11-year-olds and 55% of adults in the village are overweight. Among youths, figures indicate early alcohol and tobacco uptake and a dietary intake that is low in fruit (72%) and vegetables (80%), with most youths (84%) not meeting physical activity norms (4, 5). Furthermore, up to 16% of adults are at risk for psychosocial problems (6). A previous study in Katwijk described an intergenerational pattern of adverse health outcomes that included cardiometabolic conditions, musculoskeletal pain, and psychological distress across generations (3). Child care professionals persistently have reported low attendance at school-based prevention programs and primary care programs, and underscored the need to take parenting and the family environment into account in children's health behavioral change efforts (5).

Overweight, a sedentary lifestyle, and psychosocial stress in childhood are associated with adverse health outcomes later in life (7–9). Adverse childhood experiences and obesity are associated positively and have been demonstrated to elicit “long-lasting effects on the neural and biological systems involved in well-being, biomedical disease, social function, and psychopathology” (8, 10). Therefore, comprehensive assessment of health and psychosocial stress and uptake through early prevention programs is viewed as critical to improving children's health (11, 12). Research has found that despite the availability of preventive programs aimed at improving dietary intake, physical activity, and psychosocial well-being, attendance and adherence to these programs are low (1, 13).

Parents play a pivotal role by modeling, supporting, and guiding their children's health behaviors (14, 15). Considering that parental involvement is associated with child behavioral outcomes (16), parents' involvement in their children's behavioral change is essential (17, 18). However, despite recommendations to include parents as agents of change in health prevention (19–21), prevention programs often are directed at either changing parents' behavior or changing children and adolescents' behavior separately (22, 23), thereby ignoring the intergenerational aspect of health concerns and well-being. Therefore, these programs lack effectiveness in breaking vicious intergenerational cycles. For example, two-generation school programs that provide parents and children with high-quality preventive interventions were demonstrated to be more effective and efficient than programs that served them separately (24). Bridgett et al. (25) demonstrated that by improving parents' self-regulation, parenting behavior can be improved, stress decreased, and the familial context enhanced. A simultaneous focus on strengthening children's self-regulation also enhanced family interactions. Working on family goals elicited changes that resulted in positive well-being outcomes among children (25). Another recent study found that focusing on shared health goals could prevent adolescents from developing depressive symptoms and unhealthy or risk-taking behaviors (26).

Involving the setting in which children spend most of their time is likely to enhance health promotion efforts' long-term impact. This is particularly true in family-focused settings such as Katwijk, where professionals often have reported social problems, health behavioral norms, and low family support as barriers to changing children's food intake, physical activity patterns, and psychosocial well-being. Thus, the first step in improving children's health and well-being is to engage both children and their parents in preventive activities. To this end, we integrated an intergenerational approach in a frequently used and shared assessment tool for children's care needs in child preventive health care in the Netherlands. The tool, *Gezamenlijke Inschatting Zorgbehoeften* (GIZ), assesses children's strengths and needs regarding their health and well-being, as well as empowers them to set goals and create plans to manage their needs. The GIZ engagement tool has been demonstrated to elicit positive effects in discussing parenting and social circumstances, parent-health professional agreement, and parents' satisfaction (27). For our study, the GIZ methodology was adapted to address parents' strengths and needs concerning either changing their own behavior and/or helping their children with behavioral change.

The current study aimed to evaluate study participants' experiences and monitor this family-engagement tool's effects on families, in which children are overweight and/or experience psychosocial problems. Unlike most prevalent studies, which have focused on population-level effects, our first objective was to monitor within-family changes in physical activity, eating habits, well-being, and body mass index, as well as their adherence to behavioral change goals and plans. The second research objective was to understand how families changed their health behaviors or well-being and how they set (or failed to set) family goals and plans.

## Method

### Study Design

We followed 12 children ages 10–14 years and their parents for 12 weeks using an explanatory mixed-methods design that combined qualitative research and an n-of-1 study. N-of-1 studies are based on repeated observations within individuals or units (in this case, families) over time and are viewed as an important research method for generating scientific evidence about individuals' health or behavior, particularly when care is personalized to the individual (28). The Medical Ethical Committee of Leiden, Den Haag, Delft (P18.192), approved the study design.

### Participants

Six different care professionals recruited families to participate in a pilot study in which the professionals integrated the family-engagement tool in their routine work in the village of Katwijk: a nurse practitioner focusing on mental health problems in a general practitioner's office; a youth worker providing tailored sports advice; a behavioral scientist and a child health professional working with families at Child and Family Services; a dietitian; and a remedial teacher from a primary school. In the study design, it was estimated that each care professional would recruit five families. The inclusion criteria: children ages 10–14 and their parents participating in a child care service that focused on improving either healthy food intake, physical activity, or psychosocial well-being. The exclusion criteria: insufficient knowledge of the Dutch language and no informed consent from either parents or children to participate in the study. The care professionals recruited 25 families with children ages 10–14. After an initial phone call from the researchers to explain the study, 13 families agreed to participate. Of these, 12 started keeping journals three times a week, with both children and their parents encouraged to make journal entries. Eight families completed the journal with 20 or more data points. Three triads (father, mother, and child) and five dyads (mother and child) completed the journal study. Common reasons for dropping out included lack of time, mothers' ongoing difficulties motivating the child and/or spouse to make journal entries, and family's feelings like the questions in the journal did not apply to the family situation. The children in the families that dropped out were somewhat older than the average age (Table 1).

**Table 1:** Mean score on health behaviors and quality of life at baseline and follow-up.

	**Children**			**Mother**		
	**Dropout**	**T0**	**T = 1**	**Dropout**	**T = 0**	**T = 1**
*n* =	5	8	8	5	8	8
Age mean (SD)	11.60 (1.34)	10.50 (0.93)		41.75 (5.44)	41.71 (4.42)	
BMI mean (SD)	21.35 (4.27)	21.48 (5.07)	21.55 (5.10)	28.31 (9.98)	30.13 (5.55)	30.44 (5.29)
Vegetable intake mean (SD)	4.80 (0.84)	5.25 (0.89)	4.88 (0.99)	4.40 (0.55)	5.43 (0.79)	4.63 (0.52)
Daily exercise mean (SD)^a^	5.40 (1.14)	2.75 (2.12)	3.63 (1.69)	3.60 (1.82)	3.57 (1.27)	2.63 (1.51)
Kidscreen-27 total (max score = 135) mean (SD)	106.00 (16.22)	112.29 (7.89)	109.38 (109.38)	n.a.	n.a.	n.a.

*Intake per week; 1: Never, 2: Fewer than one time per week, 3: 1–2 days per week, 4: 3–4 days per week, 5: 5–6 days per week, 7: Every day*.

a *Days per week child > 60 min exercise, parent > 30 min exercise*.

### Family-Engagement Tool

**Figure 1:**
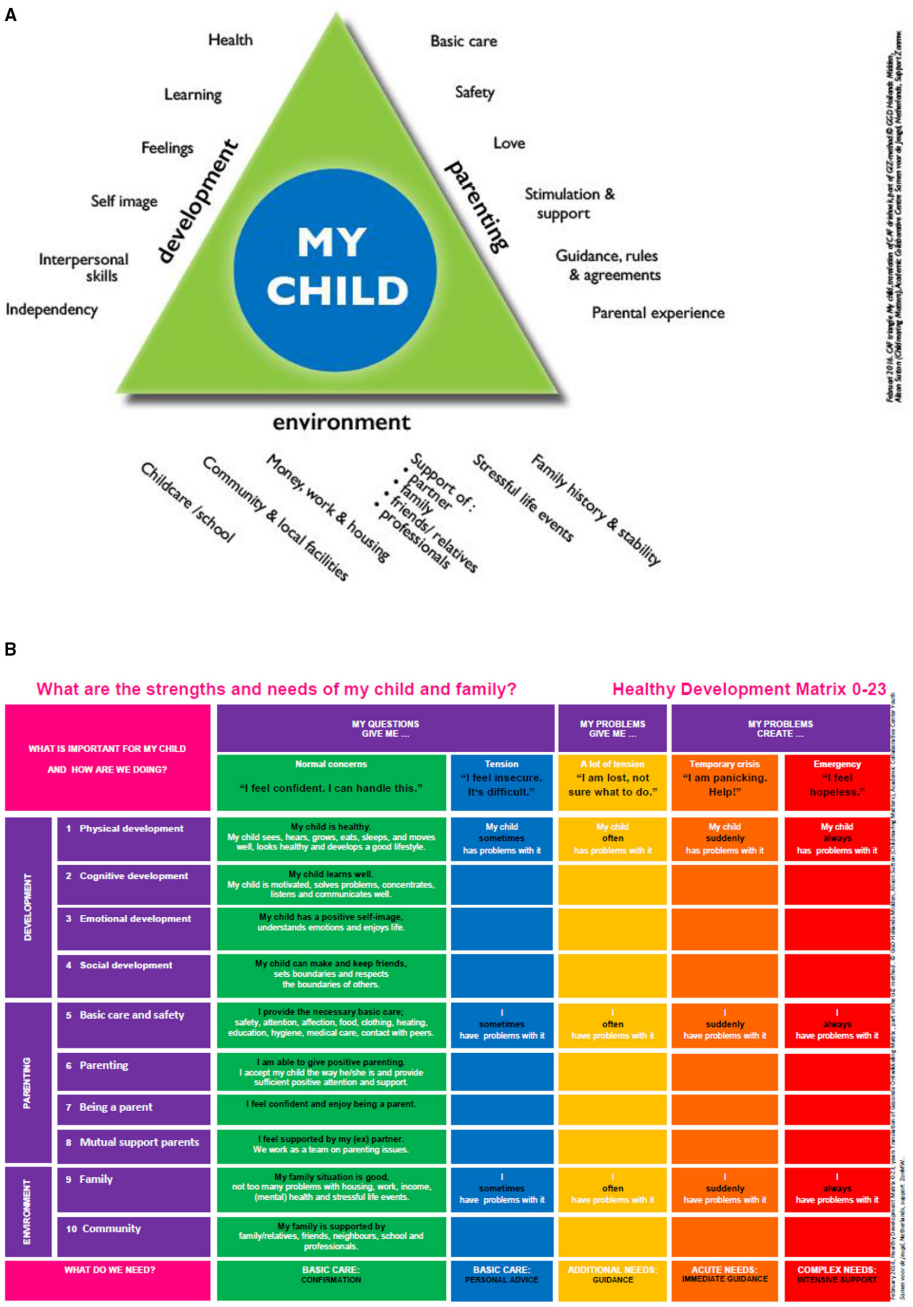
**(A)** The common assessment framework-triangle. **(B)** The healthy development matrix.

The family-engagement tool is based on the *Gezamenlijk Inschatten Zorgbehoefte* (GIZ) methodology (i.e., joint assessment of care needs), which is an integrated methodology for making shared assessments of care needs and decision-making. The GIZ methodology uses two visual, age-specific tools to structure the consultation: the Common Assessment Framework triangle (CAF) and the Healthy Development Matrix (HDM) (Figures 1A,B). To be able to tailor the tool to different target groups, different visuals were developed, e.g., age-specific visuals (parents of babies, schoolchildren, and adolescents), visuals tailored to low literacy, and visuals translated into six different languages (27). GIZ practitioners are trained through manuals, training sessions, and a support course. The GIZ methodology often is used to assess parents and/or children's needs and strengths. In this study, based on six meetings with professionals, GIZ was adapted to assess the needs and strengths of and set goals and plans for both parent(s) and child, thereby engaging the family. The family-engagement tool uses the same two visual, age-specific tools to structure GIZ consultations: the aforementioned CAF and HDM (Figures 1A,B). The method comprises three phases (introduction, analysis, and shared decision-making). During the introduction phase, the professional explains the conversation's purpose and structure, creating a common language and framework using the visual tools (CAF and HDM). Throughout the analytical phase, the professional, child, and parents discuss the family's needs and strengths in three domains: the child's development; parenting; and family and social circumstances. When care needs are identified, the professional uses the HDM tool to assess the impact and severity of care needs together with the child and parent(s). This is followed by shared goal-setting and decision-making: The child, parent(s), and professional discuss and decide which follow-up actions are necessary to secure the best outcomes for the family. In collaboration with the family, the professional develops a results-focused action and support plan that is monitored and evaluated using HDM in subsequent consultations. In our study, the professionals focused on improving dietary intake, physical activity, and psychosocial well-being in children and adolescents. They used the family-engagement tool in their regular intake procedure and were trained in using the tool with parents and children.

### Quantitative Data Collection

At baseline, families filled out the pencil-and-paper questionnaire in the presence of the researcher(s) during the initial home visit. Over a period of 12 weeks, the journals were sent on a fixed schedule thrice weekly (2 weekdays and 1 weekend day) digitally. If both parents participated, a link to the journal was sent to the father and mother's email addresses separately. Parents and children started filling out these journals online at least 3 weeks before their first visit with a health care professional in which the family-engagement tool was used. After 12 weeks, the families completed a follow-up pencil-and-paper questionnaire during the final home visit by the researcher(s). Weight and height were measured at baseline and at follow-up. During recruitment, after reading and discussing the information letters for parents and children, participants (older than 12) gave their written consent to have their data collected and analyzed.

For the baseline and follow-up, the questionnaire comprised questions in the following categories: sociodemographic characteristics (age, gender, ethnicity, education level, and employment); eating habits (daily intake of vegetables, fruit, sweets, and soda); physical activity (days per week being physically active for 30 min (parent[s])/60 min [child]); free time (daily screen time, outside play time); tobacco and alcohol use; physical well-being; and quality of life. For the health behaviors, we used the HBSC study questionnaire (29, 30). The children's quality of life and psychosomatic well-being were assessed using KIDSCREEN-27, a validated questionnaire that assesses children's quality of life based on the following categories: physical health; feelings; mood; self-reflection; spare time; family; friends; school; and money (31). A high KIDSCREEN-27 total score indicates a better quality of life. To assess the parents' quality of life, the EQ-5D-3L was used (32); this tool assesses parents' mobility, self-care, activity, pain, and mood on a scale of 1 (no difficulties) to 3 (many difficulties) for each aspect. The follow-up questionnaire comprised the same questions as the baseline questionnaire, and once again, participating members were weighed and measured. In the present study, we included questions regarding overweight, physical activity, eating behaviors, and well-being.

The journal questionnaires asked the children and parent(s) to assess their mental well-being during the previous 24 h concerning hours of sleep, energy level, stress, pain level, and sadness on a scale of 1–10. For the sleep and energy items, a higher score represented better sleep and more energy. For the items pain, sadness, and stress, a higher score represented higher pain level, more sadness, and more stress.

The journal also asked participants about vegetable intake and minutes of daily exercise. Daily vegetable intake was scored using the following scale: 1, no vegetables; 2, one serving; 3, two servings; and 4, three or more servings. Physical exercise was measured in minutes. Next, the journal asked about behavioral goals discussed with the health professionals, e.g., how easy or difficult it was to work on and achieve goals, using a scale of 0–10. The questions about goals were asked only after the visit with the care professionals.

The professionals completed a brief questionnaire after using the family-engagement tool. The questions concerned how difficult or easy it was to discuss strengths and care needs with the families (scale of 0–10), setting goals and action plans (yes/no), and referrals for children and/or parent(s) (yes/no). They were given space to elaborate on their answers. Considering that the questionnaire was anonymized with respect to the families, we could not link each professional's experiences with individual families.

### Qualitative Data

To understand the everyday dynamics of health behavioral change in these families, qualitative data were collected throughout the entire study period. For the journal part, the children and parents were invited to elaborate qualitatively on their day (thrice a week), as they were asked, “Was this in any way a special day? For example: You were ill; it is your sibling's birthday; it is a snowy day; something nice happened; something sad happened.” During home visits, at the beginning and end of the study, the second and sixth author interviewed children and parents about their upcoming or past visit with the health care professional. The face-to-face interviews were conducted in Dutch using a semi-structured interview schedule that assessed the families' experiences with the care professional, using the family engagement tool, and working on the family's health behavior goals. After participants gave their consent, the interviews were audio-taped and transcribed verbatim. Each interview lasted between 30 and 50 min.

Furthermore, we took notes from short phone and email conversations with parents, as secondary data sources, throughout the study period. For triangulation purposes, we also included anonymized evaluation forms from health care professionals in the analysis. These evaluation forms, which were filled out after each intake with the family engagement tool, assessed whether professionals set goals and made action plans.

Because obesity and psycho-social problems were “sensitive” topics in the village of Katwijk, often leading to socially desirable answers or early dropout in existing studies, we integrated arts-based data collection techniques (33). Such techniques have been used in research with children and other groups that are “hard to reach,” particularly when little research knowledge exists about the issue at stake (34).

For the face-to-face interviews at the end of the study, we built our topic guide around a descriptive vignette, using arts-based techniques. For example, to assess experiences visiting a care provider and making a family plan, we described a vignette of 10-year-old Ben and his parent, who were recently invited to see a health care professional. The vignette was tailored for each health care professional (an invitation to see a community pediatric nurse, youth worker, etc.). After reading the vignette out loud, study participants were asked to choose from three emojis or pictograms (from a sheet) to indicate how Ben and his parent would feel before and after seeing the care professional. Participants then were encouraged to elaborate on their choices, first from the perspective of the child and parent in the vignette (“Could you explain why Ben/mother/father would feel this way?”), then from their own experiences.

(“How was your experience?”). The remainder of the topic guide included questions about priorities in making a family plan and working on health behavioral goals, which also were assessed using the vignette description and sheets with visual tools. The aforementioned interviews were recorded and transcribed verbatim.

### Analyses

Descriptive statistics were used to describe the results from the baseline and follow-up questionnaires. We then conducted the analyses, following the steps for an *n* = 1 study (35). Some data points from the *n* = 1 study were missing a large majority of the answers; these 23 data points were excluded, leaving 250 data points remaining (39 pre- and 211 post-study). Variables with fewer than 5% missing values after this exclusion were imputed. Day-specific pattern data were imputed manually. For each missing value, it was assessed on which weekday this value was missing, then the average of the previous two values on this weekday and the next value on this weekday was calculated and used as the missing value.

First, we performed multi-level time-series analyses across all children and mothers to assess whether physical activity, vegetable intake, and well-being outcomes changed over time and whether outcomes changed in the period before and after consultation with the family-engagement tool. Second, we employed regression analyses with autocorrelation correction to examine changes within individual children and their mothers and fathers (when they completed the journal). Analyses were performed using SPSS (Version 25).

The qualitative journal data were analyzed using qualitative and thematic content analytic approaches (36). Then, to understand dynamics in health behavioral change within and across the individual families, the research team jointly reviewed and analyzed transcripts and secondary data, and linked these to the quantitative results. NVivo 11 was used to conduct these analyses.

## Results

In 20 weeks, five professionals used the family engagement tool 25 times with children ages 10–14, and 13 families entered the pilot study, with eight used in the main study ultimately. In what follows, we first report on the families' characteristics and their experiences with the family-engagement tool, goal-setting, and action planning. Next, we describe results on health behaviors and well-being across all families, followed by the trajectories of individual families.

Altogether, eight children and their parent(s) completed the study. More dyads (mother and child) than triads (mother, father, and child) entered and completed the study, with a total of three triads finishing the study. Six children visited a health professional for weight-related concerns, with five invited for a follow-up for a preventive health care check with the community pediatric nurse and one visiting the youth worker for tailored physical exercise. In each of these six families, there was an intergenerational pattern of overweight. The mothers' BMI varied between 29.25 and 39.67, and the BMI of the three fathers between 23.55 and 31.02.

Two children visited a health professional because of psychosocial problems. At baseline, these children had a healthy weight, as did their mothers (average BMIs of 23.62 and 24.04, respectively). When comparing the baseline with follow-up data at 12 weeks across the eight families, there were no important changes in BMI, health behaviors, quality of life, or parental concerns (Table 1).

### Experiences With the Family-Engagement Tool

The families in the study indicated that visiting a child health professional can be stressful, eliciting statements such as “You are not quite sure what will happen,” “I don't want to be the only one in my class going there,” and “I am afraid that my child will be sad because of what is discussed.” The reasons to visit the professional despite initial fears were discussed retrospectively, with advantages cited, such as “hearing that my child develops well, also has strong sides,” “finding solutions” and “the professional is quite nice.” When asked what professionals should know to be able to set goals and action plans, all the families described the importance of being aware of the emotions of children/parents, peer relations, and parenting and contextual factors (such as income). Two families did not remember using the family-engagement tool during their visit with the child health professional. In one family, the child remembered using the tool, but her mother did not.

The professionals who used the tool found it easy to discuss the child and parent strengths (mean = 8.1 [standard deviation = 0.8]), and also reported that it was relatively easy to discuss concerns regarding the child and family (mean = 7.5 [SD = 1.0]).

As for setting goals and creating action plans during the consultation, three of the children indicated that they did not set any goals. The other five set goals right after the consultation or a few weeks after the consultation. Seven of the eight mothers reported setting goals at the same time point as the child. The post-intervention interviews revealed that in many cases, there was no clear or specific goal setting or action planning during the visit with the care professional. This finding was reported particularly by the overweight children and their families. Rather than discuss goals, these families were advised to continue with the activities that the child and parents already had initiated. The professionals reported that in 29% of the consultations in which they used the family-engagement tool, no goals were set. In 65% of the consultations, professionals did not develop an action plan with the child and parents. Reasons for not setting goals were that the child and family already had started changing health behaviors, the child was doing well, or there were so many (other) concerns that weight was not the most important concern on which to focus. One family reported that it was easy to set goals, as the goal was to continue what the family already started. In another family with concerns about the child's psychosocial development, the professional reported that during the consultation using the family-engagement tool, the family, particularly the adolescent, displayed anger and resistance to change; therefore, no goals were set at that time. With another family, the parent was reluctant to discuss the child's overweight status with the child present. However, they set goals and made a plan.

### Journal Effects on Health Behaviors and Well-Being Across and Within Families

The children's physical activity mean minutes increased significantly during the period after their consultation with the health professional, compared with the period before the consultation (Table 2). Their vegetable intake did not differ significantly compared with behavior before the consultation with the family-engagement tool, nor did their hours of sleep or levels of pain, energy, or happiness. The mothers did not change their physical activity or vegetable intake levels significantly, but they felt more energetic during the period after the consultation with the family-assessment tool (Table 2).

**Table 2:** Multi-level time-series analyses of the combined effects on well-being and health behavior of children and mothers.

	**Child ***N*** = 8**			**Mother ***N*** = 8**		
	**Timepoints = 39 Mean (sd)**	**Timepoints = 211 Mean (sd)**	Effects B (95% CI)^*^	**Timepoints = 39**	**Timepoints = 211**	Effects B (95% CI)^*^
**Sleep: hours**						
Family-engagement^$^	7.96 (1.62)	8.5 (1.87)	0.40 (−0.22–1.03)	6.84 (2.13)	7.87 (1.65)	0.66 (−0.04–1.35)
Time			−0.01 (−0.04–0.02)		−0.01 (−0.11–0.09)	
**Energy level, 0–10**						
Family-engagement^$^	7.61 (1.48)	8.03 (2.26)	0.44 (−0.36–1.25)	7.19 (1.59)	7.85 (1.26)	**0.67 (0.12–1.22)**
Time			−0.01 (−0.04–0.02)		−0.02	(−0.36–0.32)
**Stress level, 0–10**	n.a.	n.a.	n.a.			
Family-engagement^$^				2.08 (2.36)	1.71 (2.30)	−0.70 (−1.45–0.05)
Time						0.02 (−0.04–0.07)
**Sadness level, 0–10**						
Family-engagement^$^	0.67 (0.85)	0.77 (1.25)	0.10 (−0.41–0.60)	0.97 (1.93)	1.15 (2.08)	−0.28 (−0.91–0.36)
Time			−0.01 (−0.04–0.02)			0.00 (−0.02–0.02)
**Pain level**						
Family-engagement^$^	1.45 (2.40)	1.14 (1.69)	−0.59 (−1.29–0.11)	0.64 (1.03)	0.68 (1.23)	−0.06 (−0.51–0.41)
Time			−0.00 (−0.05–0.04)			−0.01 (−0.03–0.02)
**Physical active, min**						
Family–engagement^$^	64.5 (48.13)	110.44 (59.39)	**26.83 (5.31–48.36)**	56.79 (62.27)	87.24 (65.97)	17.98 (−3043818.80–3043854.77)^#^
Time			0.57 (−0.68–1.81)			−0.11 (−812.07–811.86)
**Vegetable intake; spoons**						
Family–engagement^$^	2.18 (0.97)	2.16 (1.04)	0.06 (−0.32–0.44)	2.44 (1.10)	2.62 (1.02)	0.04 (−0.36–0.44)
Time			0.00 (−0.01–0.01)			−0.00 (−0.03–0.02)

* *Multi-level model: including family and timepoints as levels; n.a., not asked*.

$ *Reference group is period before the first consultation with family-engagement tool, bold = p < 0.05*.

# *Large differences in reported minutes of physical activities between mothers*.

A closer examination of the individual cases indicated that in five families, the children increased their physical activity after their consultation with a health professional. In three of these five families, physical activity increased significantly (Table 3). By relating these quantitative data to the qualitative data, we tried to understand the families' change trajectories in health behavior and well-being. To contextualize families' trajectories in the study, we used fictitious names for the children and integrated anonymized qualitative data from the journals and interviews.

**Table 3:** Regression analyses of the effect of time and pre and post first care professional visit for each of the eight families, separated for child, mother and father (when completed).

		**Sleep**	**Energy**	**Stress**	**Sadness**	**Pain**	**Physical activity**	**Vegetable intake**
		B (95% CI)^*^	B (95% CI)^*^	B (95% CI)^*^	B (95% CI)^*^	B (95% CI)^*^	B (95% CI)^*^	B (95% CI)^*^
**Family 1**								
Child	Engagement tool^$^ Time	1.26 (−1.36–3.89) 0.01 (−1.37–3.89)	**3.17 (1.34–5.01)** −0.01 (−0.05–0.04)	n.a.	0.78 (−2.08–3.64) 0.03 (−0.02–0.09)	−1.63 (−6.24–2.97) −0.01 (−0.10–0.08)	**76.62 (22.63–130.60)** −0.62 (−1.67–0.43)	0.02 (−2.16–2.19) −0.01 (−0.04–0.02)
Mother	Engagement tool^$^ Time	**2.89 (1.36–4.42**) 0.00 (−0.03–0.03)	0.21 (−1.69–2.12) −0.02 (−0.05–0.02)	−1.51 (−3.52–0.55) 0.02 (−0.02–0.07)	−2.74 (−4.45 to –1.03) 0.02 (−0.02–0.05)	1.60 (−1.01–4.20) −0.04 (−0.08–0.01)	3.05 (−79.44–85.53) 0.30 (−1.58–2.18)	**1.89 (0.65–3.13)** −0.04 (−0.07–0.01)
Father	Condition Time	−0.73 (−2.07–0.61) 0.01 (−0.03–0.03)	**−1.17 (−2.16 to –0.18)** 0.00 (−0.02–0.03)	−0.90 (−2.01–0.20) 0.01 (−0.02–0.04)	0.05 (−0.24–0.34) −0.00 (−0.01–0.01)	0.33 (−1.20–1.85) −0.02 (−0.06–0.01)	−2.52 (−73.42–68.39) −0.20 (−1.82–1.42)	0.98 (−0.06–2.02) −0.02 (−0.04–0.01)
**Family 2**								
Child	Engagement tool^$^ Time	0.67 (−1.83–3.17) −0.09 (−0.29–0.11)	−0.31 (−1.94–1.31) 0.01 (−0.12–0.14)	n.a.	0.52 (−0.31–1.35) −0.09 (−0.17–0.01)	−0.35 (−1.20–0.50) 0.00 (−0.07–0.07)	40.23 (−0.41–121.49) −0.04 (−6.60–6.51)	0.04 (−1.79–1.87) 0.02 (−0.13–0.17)
Mother	Engagement tool^$^ Time	−1.31 (−4.39–1.78) 0.23 (−0.02–0.48)	0.39 (1.32–2.11) 0.11 (−0.04–0.26)	0.97 (−0.48–2.42) **−0.16 (−0.27 to –0.04)**	−0.00 (−0.60–0.59) 0.00 (−0.05–0.05)	0.00 (−0.07–0.08) −0.07 (−0.21–0.08)	50.55 (−17.95–119.05) −2.44 (−7.96–3.08)	0.52 (−0.18–2.21) −0.06 (−0.20–0.08)
**Family 3**								
Child	Engagement tool^$^ Time	−0.73 (−4.35–2.89) −0.00 (−0.09–0.08)	−0.15 (−5.41–5.11) −0.03 (−0.15–0.10)	n.a.	**−1.84 (−3.13 to –0.55)** **−0.04 (−0.07 to –0.01)**	1.29 (−1.00–3.58) −0.03 (−0.08–0.01)	−25.41 (−119.94–69.21) 2.21 (−0.03–4.46)	0.29 (−1.15–1.73) 0.01 (−0.03–0.04)
Mother	Engagement tool^$^ Time	0.73 (−3.04–4.50) −0.04 (−0.13–0.05)	1.26 (−2.36–4.88) −0.02 (−0.08–0.05)	−0.61 (−5.39–4.17) 0.107 (−0.01–0.22)	2.37 (−2.87–7.60) −0.02 (−0.14–0.11)	0.17 (−2.63–2.97) −0.03 (−0.09–0.04)	31.37 (−66.66–129.39) −0.25 (−2.18–2.08)	−1.61 (−4.38–1.16) 0.05 (−0.00–0.10)
**Family 4**								
Child	Engagement tool^$^ Time	0.68 (−0.59–1.95) 0.03 (−0.00–0.062)	0.19 (−0.82–1.20) 0.00 (−0.02–0.03)	n.a.	0.46 (−1.19–2.12) −0.01 (−0.05–0.03)	−0.33 (1.44–0.79) 0.03 (0.01–0.06)	**71.09 (25.09–117.08)** −0.35 (−1.50–0.81)	−0.66 (−1.58–0.26) 0.01 (−0.01–0.04)
Mother	Engagement tool^$^ Time	−0.42 (−1.29–0.44) 0.01 (−0.02–0.03)	−0.17 (−0.93–0.60) −0.01 (−0.03–0.01)	−0.32 (−2.11–1.46) −0.03 (−0.07–0.02)	−0.43 (−1.16–0.30) −0.01 (−0.03–0.01)	0.02 (−0.41–0.44) 0.00 (−0.01–0.01)	−1.59 (−16.51–13.34) 0.14 (−0.21–0.48)	−0.59 (−0.02–0.02) −0.00 (−0.02–0.02)
Father	Engagement tool^$^ Time	−1.16 (−3.24–0.93) 0.03 (−0.03–0.08)	0.00 (−1.27–1.28) 0.01 (−0.02–0.04)	**−3.07 (−4.66 to** **−1.49)** 0.01 (−0.03–0.05)	−0.07 (−0.36–0.22) −0.00 (−0.01–0.00)	0.92 (−0.36–2.20) −0.03 (−0.06–0.00)	−7.59 (−24.04–8.86) −0.01 (−0.42–0.41)	−0.26 (−1.17–0.66) 0.01 (−0.01–0.03)
**Family 5**								
Child	Engagement tool^$^ Time	1.77 (−0.33–3.86) −0.06 (−0.18–0.06)	0.39 (−2.73–3.51) −0.00 (−0.19–0.18)	n.a.	0.32 (−0.63–1.28) −0.05 (−0.11–0.01)	−1.38 (−3.06–0.30) −0.05 (−0.14–0.05)	−3.13 (−57.57–51.32) 1.61 (−1.56–4.77)	0.15 (−0.40–0.70) −0.78 (−2.65–1.09)
Mother	Engagement tool^$^ Time	0.52 (−3.71–4.76) **−0.27 (−0.52–0.03)**	0.01 (2.30–2.31) −0.11 (−0.24–0.03)	0.05 (−4.65–4.75) 0.08 (−0.19–0.36)	**−3.30 (−6.22 to –0.39)** 0.10 (−0.07–0.27)	−0.24 (−0.61–0.13) **0.04 (0.02–0.06)**	20.66 (−29.21–70.52) −0.69 (−3.25–1.86)	0.42 (−1.36–2.21) 0.01 (−0.10–0.11)
**Family 6**								
Child	Engagement tool^$^ Time	−0.30 (−0.85–0.26) 0.01 (−0.01–0.03)	**1.85 (0.10–3.60)** 0.02 (−0.04–0.07)		0.75 (−0.46–1.96) −0.02 (−0.07–0.02)	**−2.90 (−5.50 to –0.30)** −0.04 (−0.13–0.06)	18.38 (−0.43–79.88) −0.36 (−2.48–1.77)	−0.92 (−2.49–0.66) 0.03 (−0.03–0.08)
Mother	Engagement tool^$^ Time	1.35 (−0.40–3.09) 0.09 (0.02–0.17)	**1.73 (0.06–3.41)** 0.00 (−0.06–0.06)	**−2.15 (−3.93–0.36)** 0.00 (−0.06–0.07)	0.15 (−0.19–0.49) 0.00 (−0.01–0.01)	−0.14 (−0.55–0.28) 0.01 (−0.00–0.03)	28.06 (−18.58–74.69) 0.02 (−1.68–1.71)	−0.48 (−1.87–0.91) 0.00 (−0.04–0.04)
**Family 7**								
Child	Engagement tool^$^ Time	**2.01 (0.91–3.11)** **−0.06 (−0.09 to –0.03)**	0.74 (−1.50–2.98) −0.03 (−0.09–0.03)	n.a.	−0.11 (−0.33–0.11) −0.00 (−0.01–0.00)	−0.07 (−0.41–0.28) −0.00 (−0.01–0.01)	1.49 (−0.3158–34.56) **−0.92 (−1.79 to –0.05)**	0.79 (−0.70–2.28) −0.02 (−0.06–0.02)
Mother	Engagement tool^$^ Time	−0.44 (−2.26–1.37) −0.03 (−0.07–0.02)	−0.72 (2.44–1.00) −0.02 (−0.06–0.03)	0.25 (−0.11–0.61) −0.01 (−0.02–0.00)	0.01 (−0.17–0.17) 0.00 (−0.00–0.01)	−0.02 (−1.74–1.70) 0.02 (−0.03–0.06)	1.91 (−22.83–26.66) 0.30 (−0.95–0.36)	−0.46 (−1.83–0.91) −0.00 (−0.04–0.03)
Father	Engagement tool^$^ Time	0.66 (−0.96–2.29) 0.02 (−0.03–0.06)	0.37 (−1.08–1.81) 0.02 (−0.02–0.05)	0.17 (−1.96–2.29) −0.07 (−0.12–0.01)	0.96 (−0.81–2.73) −0.04 (−0.00–0.00)	0.68 (−0.71–2.07) −0.02 (−0.05–0.02)	**−18.67 (−35.28 to –2.05)** 0.15 (−0.29–0.59	−0.24 (−1.71–1.23) 0.00 (−0.04–0.04)
**Family 8**								
Child	Engagement tool^$^ Time	−0.34 (−1.80–1.11) −0.03 (−0.10–0.04)	−1.36 (−3.69–0.96) 0.41 (−0.08–0.16)	n.a.	−0.35 (−2.14–1.44) 0.04 (−0.05–0.13)	0.32 (−1.64–1.01) 0.05 (−0.02–0.12)	−28.75 (−96.19–38.69) **3.47 (0.05–6.88)**	−0.34 (−1.44–0.75) 0.03 (−0.03–0.09)
Mother	Engagement tool^$^ Time	0.56 (−1.41–2.52) −0.00 (−0.10–0.10)	**1.93 (0.26–3.60)** −0.08 (−0.16–0.01)	0.18 (−2.10–2.45) −0.08 (−0.19–0.04)	0.13 (−0.32–0.59) 0.01 (−0.01–0.03)	**−1.78 (−2.95 to –0.62)** 0.01 (−0.05–0.07)	24.69 (−46.98–96.35) −0.47 (−4.10–3.16)	0.20 (−0.74–1.13) 0.01 (−0.04–0.05)

* *Adjusted for autocorrelation*,

$ *reference = period before visit with care professional, bold = p < 0.0.05; n.a., not asked to the child*.

In Family 1, 12-year-old Grace (fictitious name) and her parents consulted a practice nurse (at the general practitioner's office) because of her anxiety problems. Grace and her mother both reported that they had set goals during the consultation with the practice nurse, who referred them to a psychologist after a few sessions. During the pre-consultation phase, Grace mostly reported feeling sick. She subsequently increased physical activity significantly and felt more self-perceived energy during the period after the consultation with the practice nurse. Post-consultation, she started mentioning activities with peers, which could explain the significant increase in physical activity. Activities included “going to the beach with my friend,” “volleyball,” and “swimming pool visit with dad.” Her mother, who reported an increase in hours of sleep, mentioned visits to the practice nurse and psychologists in her journals, who provided her with tools to cope with her concerns and problems. The father mentioned proactively losing weight during the study (his weight decreased from 95 to 88 kg). He also reported feeling less energetic during the period after the consultation, but the cause of this lower energy was not clear.

In another family (Family 4), Carly (age 11) and her mother visited the community pediatric nurse, who invited them for a consultation related to the child being overweight. In the journal study, Carly indicated that she had set a goal that she found quite easy to achieve. Over time, Carly increased her minutes of physical activity significantly. Her BMI decreased slightly after 12 weeks, from 22.32 to 22.16. In the post-consultation journals, she reported participating in social activities, including “a sleepover at my friend” or “I stayed over at grandma's with my family.” However, Carly's mother indicated that she had not set a goal, which was consistent with what her father reported in his journal. Carly's mother did not recollect making a plan with the community pediatric nurse: “She always scores higher on the growth curves…. The health professional said that she will have a growth spurt soon; her weight will then decrease on its own.” The mother did not change in terms of well-being, physical activity, or vegetable intake. During the interview, she mentioned that working on her health was complicated by continuous family health issues during the study period, as recorded in the journals. For example, one of her journals said: “It's my birthday, and I have three sick children at home.” In one of his journals, Carly's father reported that he was not aware of his child's food intake: “*I often do not know what my child eats and what her goals are because I work irregularly, and 2 days a week, I am in another city (with an overnight stay) because of training*.”

In Family 8, the family visited the behavioral scientist at Youth Care Services due to concerns over Brian's (age 12) mental health. In this setting, the professional created a plan with the family, with goals for Brian and his parents, which were evaluated on a weekly basis. If the plan proved to be too difficult, the health professional discussed new coping tools and/or adapted the plan. Brian increased the number of minutes of physical activity over time (independent of the consultation). His mother reported experiencing less pain and was more energetic post-consultation.

In Family 2, Walt, an 11-year-old, joined a guided physical activity group, which focused on providing a positive sports experience to overweight children. Besides a general goal to be more physically active, the mother and child reported that they did not set a specific goal with the physical activity coach. Walt increased his minutes of physical activity (although not statistically significantly), as did his mother. On the days when the child visited the physical activity coach, his number of physical activity minutes was higher. His BMI decreased from 20.30 to 19.82.

In Family 6, Stella (age 10) and her mother increased their physical activity, albeit not significantly. Stella increased her physical activity by joining (family) activities, such as going to the beach, swimming pool visits, and participating in a march, which her mother encouraged. In the journals, her mother noted: “She has done so well” and “I am so proud of her going to the pool!” They were both significantly more energetic during the period after the consultation with the health professional. They reported that no goals were set regarding Stella's weight. Her BMI increased slightly after 12 weeks, from 25.00 to 25.72. During the interview, her mother mentioned that Stella already was physically active, ate healthy, and did not eat or drink large quantities of soft drinks or candy, making it difficult to think of what to change. Upon reflecting on her experiences with the family-engagement tool, Stella's mother spoke about feeling surprised that the health professional was not aware of the details of their family situation, which became evident during the consultation with the family-engagement tool. Her husband, Stella's father, had passed away, leaving behind a family with five young children, which was not noted in the child's file. Stella's mother stated that such information should be taken into account, as this might influence Stella's health, including health behavior, and well-being, as well as the mother's ability to implement change.

Three other children visited a child health professional regarding being overweight, but did not increase their physical activity or vegetable intake. Bella, the child from Family 7, significantly decreased physical activity minutes over time, independent of the consultation, with her BMI increasing from 27.58 to 28.16. Her father also reported fewer minutes of physical activity. During the consultation, the family and the health professional discussed a general goal to change Bella's weight from some concerns (orange zone in the HDM, Figure 1B) to no concerns (the green zone). However, Bella's mother also reported that the health professional expected a growth spurt, which was connected to the agreement to “be a little bit more careful” and to continue as they had done before.

In Family 5, Lucy (age 11) visited the child health professional for her weight, which had decreased since the last visit with the health professional. During the interview, Lucy's mother mentioned that because her daughter had lost weight, the goal was to “keep (up) the good work.” During the study period, Lucy's BMI decreased, from 25.38 to 24.70. Her father did not participate in the study. Like many other mothers in the study, Lucy's mother linked this to his long working hours: “Once he gets home, he just wants to be at peace.” This was one of the few families that remained in the study despite the mother's initial distrust of preventive screening and recollections of previous negative weight-related interactions with health professionals (as discussed during the interview).

Mabel, the child from Family 3, and her mother were invited to see the child health professional after going through several life events. Mabel's parents recently divorced, and her mother and the children had moved to a (smaller) home on the other side of town, far away from peers and the extended family. Speaking about weight immediately elicited shame and sadness, Mabel agreed to enter the study on the condition that she would not be weighed. Mabel reported a significant decrease in sadness after the consultation with the child health professional, who tailored her consultation to Mabel's fear of being weighed. The mother, child, and health professional did not really discuss a plan regarding the child being overweight, although the professional provided advice about grocery shopping habits, such as refraining from buying sweets and sugary drinks. In light of ongoing stress related to the divorce and major changes in family life, it was jointly decided to focus first on non-weight concerns. They discussed support, such as language and speech guidance and psychosocial support for the parents and child, and decided to focus on reducing the child's weight during a later stage.

## Discussion

Overall, after a consultation using the family-engagement tool, the children's physical activity improved. However, the mothers' health behavior during the study changed to a lesser degree, although they were more energetic. These results seem to be in line with extant studies' findings that family engagement and decision-making can enhance the impact of interventions that aim to improve children's health (24). However, in focusing on the individual families, we found that effects differed considerably between them. Some families seem to have altered their behavior and demonstrated changes in their well-being, explaining overall effects, while others did not. The data indicated that the family-engagement tool often was used without setting specific or family goals. Whenever goals were set, families reported more changes. Below, we discuss our findings in light of studies that have examined barriers to instigating (health) behavioral change, particularly goal setting, within families.

Our findings seem to indicate that setting specific goals and action plans can help elicit engagement in activities and (health) behavioral change in some children and parents (16). While consultations for child mental health problems led families to set goals and engage in more (everyday) activities, consultations focusing on children's overweight often did not stimulate this engagement process. The differences in goal setting and behavioral change might have been related to common perceptions of overweight in this community. In line with previous studies in Katwijk and elsewhere, mothers commonly stated that their children's weight was not related to their health behaviors, with obesity perceived as something that children “outgrow” in adolescence (1, 3, 37). The difference in engagement and goal setting between families also might reflect different drivers for visiting a health care professional. Parents generally instigated consultations related to mental health problems, while consultations related to overweight resulted from an invitation from the community pediatric nurse after a routine preventive health visit at school. Taken together, our findings confirm that differences in explanatory models for overweight and the absence of intrinsic motivation function as important barriers to health behavior change among youths and their parents (38, 39).

The family-engagement tool was developed to identify strengths and needs in children's development, parenting, and the family's social context (27). Consequently, goals and plans depend on identification of these concerns. Our study confirms that when urgent child, parenting, or contextual issues emerge, these are likely to be prioritized over goals in the physical health domain (40). In at least one of the families in this study, contextual concerns led to limited action related to the child's overweight. Our findings also are in line with those of studies that have demonstrated the complexity of truly integrating a two-generation approach in health care and community settings, with an emphasis on the prevention of overweight (41). Setting goals with multiple family members undeniably means touching upon parents' childrearing practices or their own food or physical activity habits, which can be sensitive issues for the professional to address. To be able to use the family engagement tool as intended (42), more research is needed to examine which skills are needed to navigate such complexities entailing multiple and interrelated care needs and goals.

In four families, the fathers did not participate in the study despite being actively involved in their children's lives, confirming that fathers tend to be more difficult to recruit for research and interventions (43–45). In this study, non-involvement often was linked to fathers working long hours and/or working abroad for extended times, a common pattern for men in this former fishing village (3). The two families in this study that displayed positive changes in their physical activity or well-being had fathers who were involved actively in filling out the journals. While our study cannot assess whether paternal involvement caused behavioral change in these children, the data suggest some kind of relationship between fathers' involvement and their children's health behavior. As demonstrated in other studies, most fathers in our study did not attend the care professional consultation with their wives and children, i.e., other strategies are needed to involve fathers (43).

One strength of this study is that it examined health behavior changes in both children and parents, as well as the dynamics within the family. Another strength of our mixed-methods evaluation is that it allowed for examining health behavior change processes in everyday life in a way that included both parents and children's perspectives. However, this study contained several important limitations that could have impacted the results' reliability significantly, the most important of which was sample size. Only eight families provided sufficient data for the journals, resulting in a lower power to detect an effect of the family-engagement tool. The use of repeated measurements to account for the limited number of participating families meant however that there were far more data points than families, thereby increasing the power. However, it should be noted that we had far more post-study data points than pre-study data points. To understand the value of the differences between pre- and post-family engagement consultation, we integrated a qualitative approach, allowing us to shed light on “the story” behind these figures within families.

Another limitation was the missing values, which we had to adjust for using manual calculation and imputation. Manual imputation allows room for human error from miscalculations. We maintain that in this setting, manual imputation would provide the most accurate values, as we accounted for weekly patterns and within-family differences.

Furthermore, response bias is a well-known phenomenon in self-reported data (46). Response bias can be a result of a lack of understanding, social desirability, or simply mistaken recollection of events. This limitation is present in all studies concerning ecological momentary assessments using self-reports. Given the large confidence intervals across mothers, it is likely that in our study, the question on the number of minutes of physical activity per day was subject to different interpretations. However, this differential understanding elicited less of an effect on time-series analyses of the individual families. Future research should combine self-reported data with more objective data, e.g., data retrieved through physical-activity-tracking devices or apps.

Considering that the study did not include a control group, it remains unknown whether differences in health behavior and psychosomatic well-being were due to the use of the family-engagement tool specifically or to the visits to the health care professionals in general. The journals on their own also can be viewed as an intervention, which could influence the results. As demonstrated in previous studies, families that entered and completed the entire journal study probably were somewhat different from those that did not enter or did not complete the study. We observed that families who spoke about a clustering of social and health concerns during the home visit and voiced distrust toward care services and/or brought up competing explanatory models for overweight during the initial phone call often did not enter the study or withdrew after a few days. The families included in this study were most likely those that felt more capable/able to discuss health behavior change and adhere to goals and plans. Despite this well-known bias, which is difficult to account for, we were able to follow, in a family-focused setting, how mothers juggled health behavioral changes in everyday life.

Finally, while the child care professionals were trained in the use of the family-engagement tool, and families generally were accepting of a broad assessment of strengths and needs, as well as a family-focused approach to behavioral change, it remains largely unknown whether the tool was used as intended. During the follow-up interviews, mothers or children occasionally did not recognize the family-engagement tool, and several families could not recall setting specific goals to improve health behavior. More implementation research is needed to map how the family-engagement tool is used to approach health behavioral change in a variety of child care services, particularly in working with families that report (urgent) concerns in various life domains.

To sum up, this study found that a family-engagement tool can exert positive effects on some families' health and well-being, particularly among those who feel capable of discussing their concerns and needs. However, it also demonstrated how difficult it is to engage families in health behavior change in the face of care needs in other life domains. Therefore, family-engagement approaches could focus more on how to develop and integrate attainable goals and plans for multifaceted health problems, as in the case of childhood obesity, e.g., by combining goals and strategies in different life domains and for different family members. In identifying the intricacies of family-focused health promotion, child care professionals' education needs to incorporate skills training for goal setting and action planning in the face of complex and multifaceted health problems.

## Data Availability Statement

The raw data supporting the conclusions of this article will be made available by the authors, without undue reservation.

## Ethics Statement

The studies involving human participants were reviewed and approved by Leiden Medical Ethical Committee Leiden Den Haag Delft. Written informed consent to participate in this study was provided by the participants' legal guardian/next of kin.

## Author Contributions

MC and MNS conceptualized and designed the study, collected data, carried out the analyses, drafted the initial manuscript, and revised the manuscript. AO collected the data, carried out the initial analyses, and revised the manuscript. NW, LS, and MCES collected the data and revised the manuscript. RR conceptualized and designed the study and revised the manuscript. All authors contributed to the article and approved the submitted version.

## Funding

This study was founded by a grant provided by the Foundation NutsOhra.

## Conflict of Interest

The authors declare that the research was conducted in the absence of any commercial or financial relationships that could be construed as a potential conflict of interest.

## Publisher's Note

All claims expressed in this article are solely those of the authors and do not necessarily represent those of their affiliated organizations, or those of the publisher, the editors and the reviewers. Any product that may be evaluated in this article, or claim that may be made by its manufacturer, is not guaranteed or endorsed by the publisher.
